# Identify-Isolate-Inform: A Tool for Initial Detection and Management of Zika Virus Patients in the Emergency Department

**DOI:** 10.5811/westjem.2016.3.30188

**Published:** 2016-04-04

**Authors:** Kristi L. Koenig, Abdulmajeed Almadhyan, Michael J. Burns

**Affiliations:** * University of California Irvine Medical Center, Department of Emergency Medicine, Center for Disaster Medical Sciences, Orange, California; † University of California Irvine Medical Center, Department of Emergency Medicine and Department of Medicine, Division of Infectious Diseases, Orange, California; ‡ Qassim University, Department of Emergency Medicine, Saudi Arabia

## Abstract

First isolated in 1947 from a monkey in the Zika forest in Uganda, and from mosquitoes in the same forest the following year, Zika virus has gained international attention due to concerns for infection in pregnant women potentially causing fetal microcephaly. More than one million people have been infected since the appearance of the virus in Brazil in 2015. Approximately 80% of infected patients are asymptomatic. An association with microcephaly and other birth defects as well as Guillain-Barre Syndrome has led to a World Health Organization declaration of Zika virus as a Public Health Emergency of International Concern in February 2016. Zika virus is a vector-borne disease transmitted primarily by the Aedes aegypti mosquito. Male to female sexual transmission has been reported and there is potential for transmission via blood transfusions. After an incubation period of 2–7 days, symptomatic patients develop rapid onset fever, maculopapular rash, arthralgia, and conjunctivitis, often associated with headache and myalgias. Emergency department (ED) personnel must be prepared to address concerns from patients presenting with symptoms consistent with acute Zika virus infection, especially those who are pregnant or planning travel to Zika-endemic regions, as well as those women planning to become pregnant and their partners. The identify-isolate-inform (3I) tool, originally conceived for initial detection and management of Ebola virus disease patients in the ED, and later adjusted for measles and Middle East Respiratory Syndrome, can be adapted for real-time use for any emerging infectious disease. This paper reports a modification of the 3I tool for initial detection and management of patients under investigation for Zika virus. Following an assessment of epidemiologic risk, including travel to countries with mosquitoes that transmit Zika virus, patients are further investigated if clinically indicated. If after a rapid evaluation, Zika or other arthropod-borne diseases are the only concern, isolation (contact, droplet, airborne) is unnecessary. Zika is a reportable disease and thus appropriate health authorities must be notified. The modified 3I tool will facilitate rapid analysis and triggering of appropriate actions for patients presenting to the ED at risk for Zika.

## INTRODUCTION

Following closely after the 2014 Ebola outbreak, due to concerns for rapid spread and a link to birth defects, the World Health Organization (WHO) declared Zika virus a Public Health Emergency of International Concern (PHEIC) on February 1, 2016, making it only the fourth time in history for such a declaration.^1^ A PHEIC is defined as an extraordinary event which is determined to include the following:

1. Constitute a public health risk to other states through the international spread of disease, and
2. Potentially require a coordinated international response.

It implies a situation that is –

1. Serious, unusual or unexpected,
2. Carries implications for public health beyond the affected state’s national border, and
3. May require immediate international action.

WHO declared prior PHEICs for the April 2009 H1N1 pandemic, in May 2014 with the resurgence of polio after its near-eradication, and in August 2014 in response to the Ebola outbreak in West Africa.

The Zika declaration is unprecedented as it is the first time that a vector-borne disease, i.e. an infection that is not generally transmitted from person-to-person, has been deemed a PHEIC. Vector-borne diseases, including malaria and dengue, account for more than 17% of all infectious diseases and cause more than one million deaths annually.^2^ While diseases spread by vectors are among the most complex of all infectious diseases to prevent and control, identification and management in the emergency department (ED) setting differs dramatically from preparation for other infectious diseases such as Ebola, measles, and Middle East Respiratory Syndrome (MERS), each of which require immediate implementation of various types of isolation (contact, airborne, and droplet, respectively).

**Figure 1:**
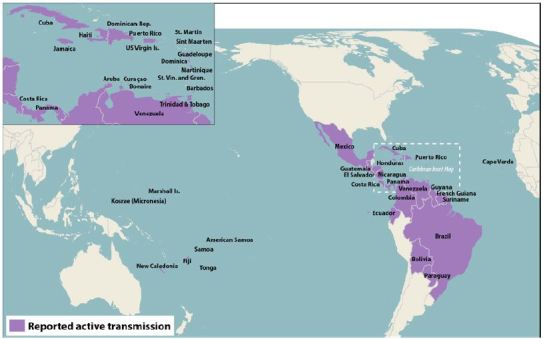
Countries with confirmed Zika cases as of April 4, 2016.^*^ ^*^Source: http://www.cdc.gov/zika/geo/active-countries.html.

Zika virus is a RNA flavivirus related to dengue, yellow fever, West Nile, and Japanese encephalitis viruses, but not to chikungunya, which is a togavirus. It was first isolated in 1947 from the serum of a febrile sentinel rhesus monkey kept in a cage on a wooden platform in the tree canopy of the Zika forest in Uganda, in which the investigators were studying yellow fever transmission.^3^ The following year it was isolated from Aedes africanus mosquitoes in the same forest. Sporadic human cases were reported from Africa and Southeast Asia over the next several decades. The first recognized outbreak occurred on Yap Island in the Federated States of Micronesia in 2007, in which an estimated 73% of the population greater than three years of age was infected and 80% of infections were subclinical. A larger outbreak then occurred in French Polynesia in 2013–2014 affecting 66% of the population, with spread to other South Pacific islands in 2014. In the French Polynesia outbreak, there was an associated large increase in the reported incidence of Guillain-Barre syndrome (GBS). A case-control study of this outbreak found a GBS attack rate of 0.24 per 1,000 Zika virus infections, with GBS developing rapidly after the onset of infection with a generally favorable outcome.^4^ Finally, the outbreak in the Western Hemisphere, which began in northeastern Brazil in 2015, with explosive spread to many countries and territories, has infected more than one million persons (Figure 1). After microcephaly was reported in the Zika virus epidemic in South America, a retrospective analysis of the French Polynesia outbreak found that the number of microcephaly cases associated with Zika virus infection was 95 (95% CI [34–191]) per 10,000 women infected in the first trimester, which is about 50 times higher than the baseline rate in the population.^5^ Zika virus RNA can be detected in amniotic fluid as well as in brain tissue of fetuses with microcephaly at a time when the maternal serum and urine are negative for RNA, supporting the association between Zika virus infection and microcephaly.^6^ As with all emerging infectious diseases,^7^ healthcare workers must keep up to date with information about how to detect and manage Zika virus.

This paper describes the adaptation of the identify-isolate-inform (3I) tool (initially developed for Ebola virus disease^8^,^9^ and modified for measles^10^ and Middle East Respiratory Syndrome (MERS))^11^ for use in the detection and management of potential Zika virus patients presenting to the ED, including women who are pregnant or contemplating pregnancy, and their partners. Using this novel 3I tool, emergency physicians will be better prepared to detect and manage patients presenting to the ED with concerns about Zika virus.

## CLINICAL PRESENTATION

Zika virus is of particular concern to women who are pregnant or considering becoming pregnant (and their partners) as it has been detected in fetuses, amniotic fluid, and semen. These patients, especially if they have been in, or are considering travel to, areas with documented Zika transmission, may present to the ED requesting information and testing.

### Signs and Symptoms

**Figure 2:**
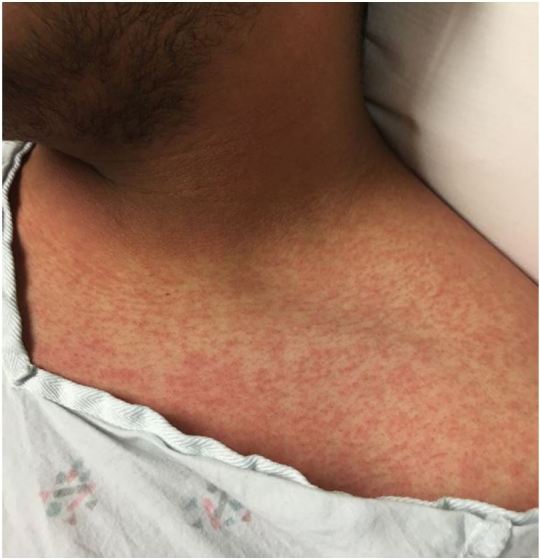
Typical maculopapular rash of Zika virus.^*^ ^*^Photograph courtesy of Dr. David C. Pigott, University of Alabama at Birmingham.

Zika infection is asymptomatic in 80% of cases. When symptomatic, it is typically described as a mild dengue-like illness, manifested by two or more of the following: sudden onset fever, conjunctivitis, arthralgias, and maculopapular rash (Figure 2). Myalgias and headache are also common. The WHO interim case definition describes suspected, probable and confirmed cases.^12^ Unlike in dengue, death from acute Zika virus infection is rare, but has been reported in a child with sickle cell disease in Colombia.^13^ The incubation period for Zika virus after a person is bitten by an infected mosquito ranges from 2–7 days, and may be up to 14 days. This is the reason for screening for risk factors within 14 days of symptom onset. The incidence of GBS or microcephaly appears to be similar between symptomatic and asymptomatic cases.

### Transmission

Zika virus is transmitted by the bite of the Aedes aegypti mosquito and likely also Aedes albopictus. Mosquito bites account for the vast majority of infections, and thus prevention from bites is the principle protective measure. In addition, Zika has been detected for prolonged periods in semen and documented cases of sexual transmission are increasing.^14^,^15^ Intrauterine/perinatal transmission also occurs. Furthermore, laboratory exposure and other blood-borne transmissions are possible; thus, blood banks have developed screening and protection criteria to mitigate spread.^16^ Theoretically, Zika virus could also be contracted from infected breast milk or organ/tissue transplants.

### Emergency Department Patient Evaluation

Patients should be assessed for pregnancy as well as for neurological symptoms and signs indicating possible GBS. While the situation is rapidly evolving, as of March 2016, laboratory testing is only available through the Centers for Disease Control and Prevention (CDC) and several state and territory health departments. Generally, testing is not recommended for asymptomatic persons, with the exception of pregnant women 2 to 12 weeks after travel to areas with ongoing Zika virus transmission.^17^ In symptomatic patients, serum should be obtained for polymerase chain reaction (RT-PCR), IgM, and IgG if within seven days of symptom onset. If more than seven days has elapsed, only IgM and IgG should be assessed; RT-PCR is not indicated.^18^ Note that serological cross reactivity may occur between Zika and other flaviviruses (e.g., dengue, yellow fever, St. Louis encephalitis, Japanese encephalitis, West Nile). Public health departments may decline to test if the clinical scenario is not suggestive of Zika virus or associated complications. In particular, due to the high cross-reactivity with related flaviviruses, serologic test interpretation is complex and can be difficult, leading to false positive results. Conversely, a negative Zika IgM or RT-PCR test result does not rule out Zika virus infection. Emergency clinicians should consult with public health experts to assist with interpretation of test results.

### Differential Diagnosis

The differential for Zika virus infection is broad as many diseases present similarly. In patients who have traveled to, or lived in, an area with ongoing Zika transmission within 14 days, chikungunya and dengue viruses, as well as malaria, leptospirosis, and rickettsial infections, are among the other infections that should be considered. Co-infection with dengue and Zika has been reported. It is likely that some travelers to Zika endemic areas will be found to be co-infected with other pathogens acquired from arthropod bites, especially malaria. Distinguishing these infections from one another based on clinical symptoms and signs can be difficult, but malaria is not typically associated with rash or conjunctivitis.

### Treatment

Treatment for Zika virus infection is entirely supportive as no specific antiviral therapy is yet available. Aspirin and nonsteroidal anti-inflammatory drugs should be avoided until dengue can be ruled out, to reduce the risk of hemorrhage. As noted, the vast majority of cases (approximately 80%) are asymptomatic; most of the remainder of patients have self-limited illnesses, with symptom resolution within seven days.^19^ Patients who develop GBS may require intensive care, including mechanical ventilation. Pregnant women who test positive for Zika virus should be offered ultrasound assessments every 3–4 weeks to assess for microcephaly with consideration for amniocentesis for Zika virus RNA testing on amniotic fluid. Fetal microcephaly is often not detectable in the first trimester of pregnancy but is most easily diagnosed in the late second and early third trimester. In some cases, it can be missed on prenatal ultrasound and not detected until after birth.

### Prevention

While under development, as of early 2016, no vaccine against Zika virus exists. Avoidance of travel to Zika-endemic areas or protection against mosquito bites is the best prevention. Mosquitoes that spread Zika bite mostly during the daytime. Preventative measures include the following:

1. Use approved insect repellents (DEET and permethrin are safe and effective for pregnant women when used in accordance with the product label)
2. Use window and door screens, or stay in air-conditioned buildings
3. Wear long-sleeved shirts and long pants
4. Use a mosquito bed net.

For patients with known or suspected Zika virus, prevent spread to others by avoiding mosquito bites (as this could infect the mosquito, which could then bite and spread the virus). Furthermore, infected men should take measures to avoid sexual transmission of virus to their partners by abstaining, using condoms, and refraining from anal intercourse and fellatio. Only male to female sexual transmission has been reported as of early 2016.

### Patient Disposition

Admission criteria for patients who are at risk for Zika virus are similar to those for any other patient. Most patients can be managed conservatively as outpatients; however, those with complications such as GBS may require hospitalization.

### Identify-Isolate-Inform

**Figure 3:**
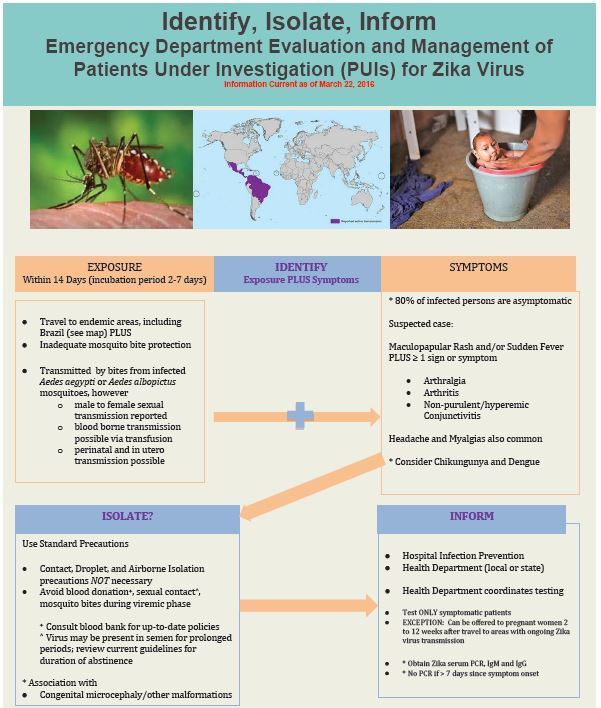
Koenig’s identify-isolate-inform tool adapted for Zika virus.

The identify-isolate-inform (3I) tool, initially developed for Ebola virus disease and subsequently adapted for measles and MERS, can be modified for the ED evaluation and management of patients under investigation for Zika (Figure 3). As most cases of Zika virus are mild and self-limited and would not be contagious in the ED setting, the tool does not need to be immediately applied on initial patient contact (as it would be for highly contagious infectious diseases).

The first step in using the Zika 3I tool is to identify patients with a possible Zika exposure within 14 days of symptom onset. This involves taking a travel history and assessing whether the patient was bitten by mosquitoes or has had sex with a patient with Zika virus. If the patient lives in, or has traveled to, areas with ongoing Zika virus transmission within two weeks, assess whether two or more of the following symptoms are present: sudden fever, maculopapular rash, arthralgias, and conjunctivitis. If the epidemiologic and symptom screens are positive, or if a pregnant patient requests testing within 2 to 12 weeks after potential exposure, investigate for the presence of Zika virus by serologic (antibody) testing.

While every patient presenting to the ED should be assessed for the potential to transmit disease (the “vital sign zero” concept)^20^, if only arboviruses, malaria or rickettsia are being considered in the differential diagnosis, isolation is not necessary. As with all patients, standard precautions should be maintained; however, contact, droplet and airborne precautions (as would be needed for selected other infectious diseases) are not required. Isolation is NOT necessary for Zika (due to its transmission characteristics, e.g. which are different from MERS and Ebola).

The second “I” in the 3I tool can be considered to be “investigate” rather than “isolate” if diseases that are contagious from person-to-person are not being considered. “Isolate,” however, remains a useful term to remind the healthcare providers who initially assesses the patient to consider whether isolation precautions are needed, as patients may present with similar symptoms associated with other infectious diseases that are contagious from person to person. In addition, to avert acquiring disease, people should “isolate” from mosquito vectors to prevent infection and, if infected, to prevent potential transmission to others.

Patients presenting in high-risk areas of the U.S. should be advised to use precautions such as staying indoors in an air-conditioned environment and wearing long-sleeved shirts and long pants. On March 16, 2016, the U.S. National Center for Atmospheric Research published a public release on potential Zika virus risk estimated for 50 U.S. cities indicating that “summertime weather conditions are favorable for populations of the mosquito along the East Coast as far north as New York City and across the southern tier of the country as far west as Phoenix and Los Angeles.”^21^ Thus, there is a heightened concern for an increased incidence of Zika virus in the U.S.

The final action of the tool is to “inform.” On January 21, 2016, U.S. government regulations authorized CDC to receive case notifications for arboviral illnesses and Zika virus, adding these entities to the list of nationally notifiable infectious diseases. In addition to notifying the hospital infection prevention and control team, emergency physicians should ensure that suspected Zika cases are reported to appropriate health authorities. Patients who do not meet medical criteria for admission should be informed about measures to take to limit the risk of virus transmission, e.g. to avoid sexual activity, mosquito bites, and blood donation.

### Relevance to Emergency Medicine

Zika virus disease will likely have substantially less direct impact on ED operations than other emerging infectious diseases such as the 2009 H1N1 influenza epidemic and 2014 Ebola virus disease outbreak. Nevertheless, due to worldwide media attention and an evolving situation, it is important for emergency personnel to be familiar with the basic principles of assessment and management of Zika virus. In particular, women who are pregnant (or considering pregnancy) and have been in (or are considering travel to) areas with ongoing Zika virus transmission, as well as women who have had sex with a male who has travelled to a region of endemic transmission, may present to the ED seeking testing and other advice. CDC issued an unprecedented travel advisory for pregnant women^22^, and health authorities have recommended that women living in affected areas delay pregnancy.^23^ In addition, patients may seek information on the potential for development of microcephaly and other birth defects and the possibility of sexual transmission of Zika virus. It is possible that in the future Zika virus will be added to the list of “TORCH” infections, which include toxoplasmosis, other (syphilis, varicella-zoster, parvovirus B19), rubella, cytomegalovirus (CMV), and herpes infections, that are associated with congenital anomalies.

Patients requiring emergency blood transfusions will need to be counseled about the risks and what measures have been taken to prevent them from contracting Zika virus. Pregnant women are likely to be particularly concerned about receiving blood. Finally, there is the potential for shortages to the blood supply due to a decrease in eligible donors, particularly as Zika virus becomes more widespread over time and during warmer weather.

### Additional Considerations

As with all contagious infectious diseases, the question of when to use the public health tools of quarantine and isolation is critical.^24^,^25^ While at least one U.S. politician has suggested that patients returning home from areas with ongoing Zika virus transmission such as Brazil should be quarantined, there is no scientific basis for this approach.^26^,^27^ As noted previously, Zika virus is not readily transmitted from person to person, either before or after symptom onset. Caution is needed to avoid negative psychosocial consequences like stigmatization such as was seen in the 2014 Ebola virus disease outbreak.^28^,^29^

Zika virus has a massive economic impact in many sectors, including healthcare. Thus, there may be a shift of resources away from other critical areas of healthcare operations and research. For example, while not approved, in the U.S. alone, President Obama requested 1.8 billion USD in emergency Zika funding.^30^

## CONCLUSION

Zika is an emerging infectious disease that has gained worldwide attention in large part due to its association with fetal microcephaly and rapid global spread. As with any novel infection, it is important not only to identify and treat individual patients, but also to protect healthcare providers and the public health. The identify-isolate-inform (3I) tool is an instrument that can be used real-time on the front lines to rapidly detect and manage patients at risk for Zika virus disease presenting to the ED. Use of the 3I tool will assist emergency physicians in performing rapid and appropriate screening and management and counseling for patients concerned about Zika virus.
